# Does formative assessment help students to acquire prescribing skills?

**DOI:** 10.1007/s00228-023-03456-w

**Published:** 2023-02-22

**Authors:** L. S. Kalfsvel, L. E. J. Peeters, K. Hoek, C. Bethlehem, I. H. van der Sijs, P. H. M. van der Kuy, W. W. van den Broek, J. Versmissen, F. van Rosse

**Affiliations:** 1 Erasmus MC, University Medical Center Rotterdam, Hospital Pharmacy, Rotterdam, The Netherlands; 2 Erasmus MC, University Medical Center Rotterdam, Institute of Medical Education Research Rotterdam, Rotterdam, The Netherlands; 3 Erasmus MC, University Medical Center Rotterdam, Department of Internal Medicine, Rotterdam, The Netherlands

**Keywords:** Formative assessment, Prescribing, Education, Pharmacotherapy, Medical student, Medical education

## Abstract

**Purpose:**

Formative assessments can help motivate students and ease students’ learning through feedback. There is a pressing need for improvement of clinical pharmacotherapy (CPT) education since junior doctors make many prescribing errors. The aim of this study was to determine whether a formative assessment with personalized narrative feedback helps medical students to increase their prescribing skills.

**Methods:**

This retrospective cohort study was conducted among masters’ medical students at Erasmus Medical Centre, The Netherlands. Students made a formative and a summative skill-based prescription assessment, both during clerkships as part of their regular curriculum. Errors in both assessments were categorized by type and possible consequence and compared with each other.

**Results:**

A total of 388 students made 1964 errors in the formative assessment and 1016 in the summative assessment. Most improvements after the formative assessment were seen for mentioning the weight of a child on the prescription (*n* = 242, 19%). Most new and repeated errors in the summative assessment were missing usage instructions (*n* = 82, 16% and *n* = 121, 41%).

**Conclusions:**

This formative assessment with personalized and individual narrative feedback has helped students to increase the technical correctness of their prescriptions. However, errors repeated after the feedback were predominantly errors showing that only one formative assessment has not yet enhanced the clinical prescribing enough.

**Supplementary Information:**

The online version contains supplementary material available at 10.1007/s00228-023-03456-w.

## Introduction

It has long been known that formative assessments not only assess students but can also help motivate students and assist students’ learning through feedback [1]. Formative assessments give the student feedback on their progress, without expressing this with grades. Opposite are summative assessments, which evaluate student learning, often by grading. Formative assessments for medical students have for example been proven to increase scores on summative assessments in pathophysiology [2] and to encourage them to learn epidemiology [3].

However, for a formative assessment to reach these goals there are several requirements. Firstly, these assessments should focus on learning and happen in a safe environment [4]. Furthermore, to enhance students’ learning through formative assessments, giving constructive feedback is required [5, 6].

There is a pressing need for improvement in clinical pharmacotherapy (CPT) education. Junior doctors make many prescribing errors [7] which can lead to patient complaints, avoidable side effects, hospital admissions, and even death [8, 9]. There have been a lot of new CPT education interventions [10], and CPT has been taught in many different ways [11], but none has had the desired extensive results in reducing prescribing errors. In the clinical setting, it has been shown that feedback from pharmacists on prescribing can effectively reduce prescribing errors [12, 13] and have a positive influence on prescribing behavior [14–17]. However, the use of feedback on prescribing, in the form of a formative assessment, as a teaching method for clinical pharmacotherapy has not yet been studied.

The aim of this study was to determine whether a formative assessment, including personalized narrative feedback, helps medical students to increase their prescribing skills based on the errors that were made in the formative and the summative prescribing assessment. The hypothesis was that the prescribing errors made in the formative assessment and on which narrative feedback was provided would appear less in the summative assessment. Furthermore, we hypothesized that errors made in the summative assessment were less severe than the errors made in the formative assessment.

## Methods

At Erasmus Medical Center, Rotterdam, The Netherlands, students make a formative skill-based prescription assessment during the fourth year of their medical curriculum in the online environment Pscribe [18]. Students may choose a time and place in the first two educational weeks prior to their surgery clerkship to make this digital assessment. During this assessment, students answer six knowledge and application questions with immediately shown feedback, followed by two case-based prescriptions for primary care patients or patients in an outpatient clinic (see appendix 1 for an example of the assessment). A CPT teacher assesses these case-based prescriptions. The students receive standardized feedback on the knowledge- and application questions and personalized feedback on the prescriptions. Students do not receive a grade for this assessment.

In their fifth year of their medical curriculum, students make the Dutch National Pharmacotherapy Assessment [19]. This assessment is a knowledge-based assessment consisting of sixty multiple-choice questions on pharmacotherapy.

**Fig. 1:**
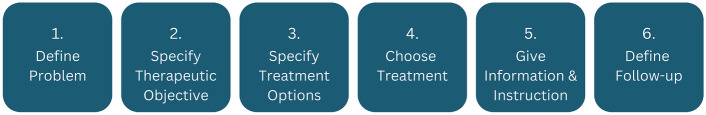
WHO six-step model

At the end of their medical curriculum, students make a summative skill-based prescription assessment (see Appendix 1 for an example of the assessment). The summative assessment is taken in the same online environment as the formative assessment; however, it is in an exam setting with a fixed time and place and supervisors. Students have to write similar case-based prescriptions, for primary care patients or patients in an outpatient clinic, as in the formative assessment. This summative assessment consists of four case-based prescriptions to write, compared to two in the formative assessment. For one of the case-based prescriptions, students need to complete a WHO six-step model, see Fig. 1 [20]. For this six-step model, students can score insufficient, sufficient, or well done per single step. Since the summative assessment is almost 2 years later in the curriculum than the formative assessment, students have acquired more knowledge. Therefore, the cases asked in the summative assessment are slightly more difficult compared to the formative assessment. In preparation for the summative assessment, students can revisit the previously given feedback on their formative assessment in their P-scribe portfolio and can choose to do a practice test.

This retrospective cohort study was conducted among masters’ medical students at Erasmus Medical Center, Rotterdam, The Netherlands. Master students who took their summative prescribing assessment between 27 July 2020 and 4 October 2021 were included. Due to the disrupted educational program as a result of the COVID-19 pandemic, the inclusion period was extended to October 2021 instead of the originally planned July 2021. Data on the formative as well as their summative assessment were extracted from the digital program P-scribe. The data extracted from P-scribe included the teachers’ feedback given during the assessment. If, during the correction of the assessment, teachers missed errors, these missed errors were not added to the dataset.

Students were excluded when either one of the assessments was not available or if feedback from the teacher was absent. Only the first attempts of the assessments were included, meaning that re-sit assessments were excluded.

Prior to the study, each student had made a personal account in the program P-scribe for educational purposes. With the registration in P-scribe, students consented to have their data saved and used for research. We coded student data to insure anonymity. The review of the research proposal by the Medical Ethics Committee Erasmus MC determined that the Medical Research Involving Human Subjects Act was not applicable to this research.

### Categorization of errors

From the teachers’ feedback extracted from P-scribe, we categorized the errors into the type of errors and possible consequences. The categorization of the type of errors (see Table 1) was based on previous studies and the Erasmus Medical Center guidelines to report an incident [7, 21–24]. The classification of the National Coordinating Council for Medication Error Reporting and Prevention (NCC MERP) was used to categorize the possible consequences of the errors [25]. All errors were categorized with the expert opinions of a medical doctor and a pharmacist. In complex cases, the error was discussed with an independent pharmacist, an internal medicine physician, and a CPT teacher until consensus was reached.

**Table 1:** Error types

**Administrative errors**	Missing patient information Error in Dutch Opium Law Non-existing drug dose Error in the structure of prescription
Inadequate information	No weight of child No indication stated when necessary No concentration or dosage stated No dosage form stated No amount to supply stated Missing maximum use Dose not measurable (e.g., 3.67 ml) Wrong usage instructions Missing usage instructions No “with controlled release” stated with the drug name when prescribed as a “with controlled release” product No duration of treatment stated
Wrong drug dose	Dose too low/high
Wrong drug interval	Incorrect drug interval “With controlled release drugs” prescribed in an interval as if not “with controlled release”
Wrong dosage form	Incorrect or less than a desirable dosage form
Wrong prescribed amount	Insufficient prescribed to finish treatment (e.g., student prescribed amoxicillin/clavulanic acid three times a day for 5 days, but only prescribes 10 tablets) Insufficient prescribed which makes the prescription patient unfriendly (e.g., student prescribed only one sildenafil tablet). Too much prescribed for newly started chronic drugs (e.g., enalapril for more than 15 days) Too much prescribed for necessary treatment (e.g., nystatin 300 ml, while 100 ml is sufficient)
Wrong drug	Wrong drug

### Repeated errors

We checked the pattern of errors for each student. We categorized all errors into three categories, namely errors which were made in the formative assessment but not in the summative assessment, errors which were only made in the summative assessment, and errors which were made in both assessments. For this analysis, if an error of the same error type was made multiple times by the same student in the same assessment, these were counted as one.

### Questionnaire on the preparation of student

To study the use of the feedback on the formative assessment in the preparation for the summative assessment, we have sent an online questionnaire to all students who took their summative prescribing assessment between 17 May 2021 and 4 October 2021 through e-mail 2 weeks after completing the assessment. At that point in time, their result on the summative assessment was not yet known. The questionnaire consisted of four questions regarding the preparation for the summative assessment. To compare students who completed the questionnaire and the students who did not, the scores of all students on the Dutch National Pharmacotherapy Assessment were used.

Data was analyzed using IBM SPSS statistics 28.0 [26]. An independent *T*-test was done to test for comparability between the group of students who completed the questionnaire and the other students. We used a *χ*^2^ test to study the differences in error types and to study whether errors were repeated or not. Further data analysis was done with descriptive statistics.

## Results

A total of 452 students made at least one of the assessments during the selected period. From 388 of these students, information on both assessments was available. These students had an average age of 25 years, and 67% were female.

On average, these students made 1.9 errors *less* [1.7–2.0 95% CI, *P* < 0.001] per prescription in the summative assessment compared to the formative assessment (see Table 2).

**Table 2:** Consequences of errors in formative and summative assessment

	**Formative assessment (two prescriptions)**		**Summative assessment (four prescriptions)**	
	Total (*n* and %)	Per prescription (*n* = total divided by two prescriptions)	Total (*n* and %)	Per prescription (*n* = total divided by four prescriptions)
**Total number of errors**	1964	2.5 (SD 1.3)	1016	0.6 (SD 0.5)
**Category B errors**	1018 (51.8%)	509	220 (21.7%)	55
**Category C errors**	375 (19.1%)	187.5	455 (44.8%)	114
**Category D errors**	422 (21.5%)	211	251 (24.7%)	63
**Category E errors**	148 (7.5%)	74	90 (8.9%)	22.5
**Category I errors**	1 (0.1%)	0.5	0 (0%)	0

Errors in formative versus summative assessment, % error category/total errors per assessment. Category B: an error occurred but the error did not reach the patient. Category C: an error occurred that reached the patient but did not cause the patient harm. Category D: an error occurred that reached the patient and required monitoring. Category E: an error occurred that might have resulted in temporary harm. Category I: an error occurred that would have resulted in patient death

In the formative assessment, the majority of errors (*n* = 1018, 51.8%) was category B (an error occurred but did not reach the patient) error. In the summative assessment, the majority of errors (*n* = 455, 44.8%) was category C (an error reached the patient but did not cause harm) error.

### WHO six-step

**Fig. 2:**
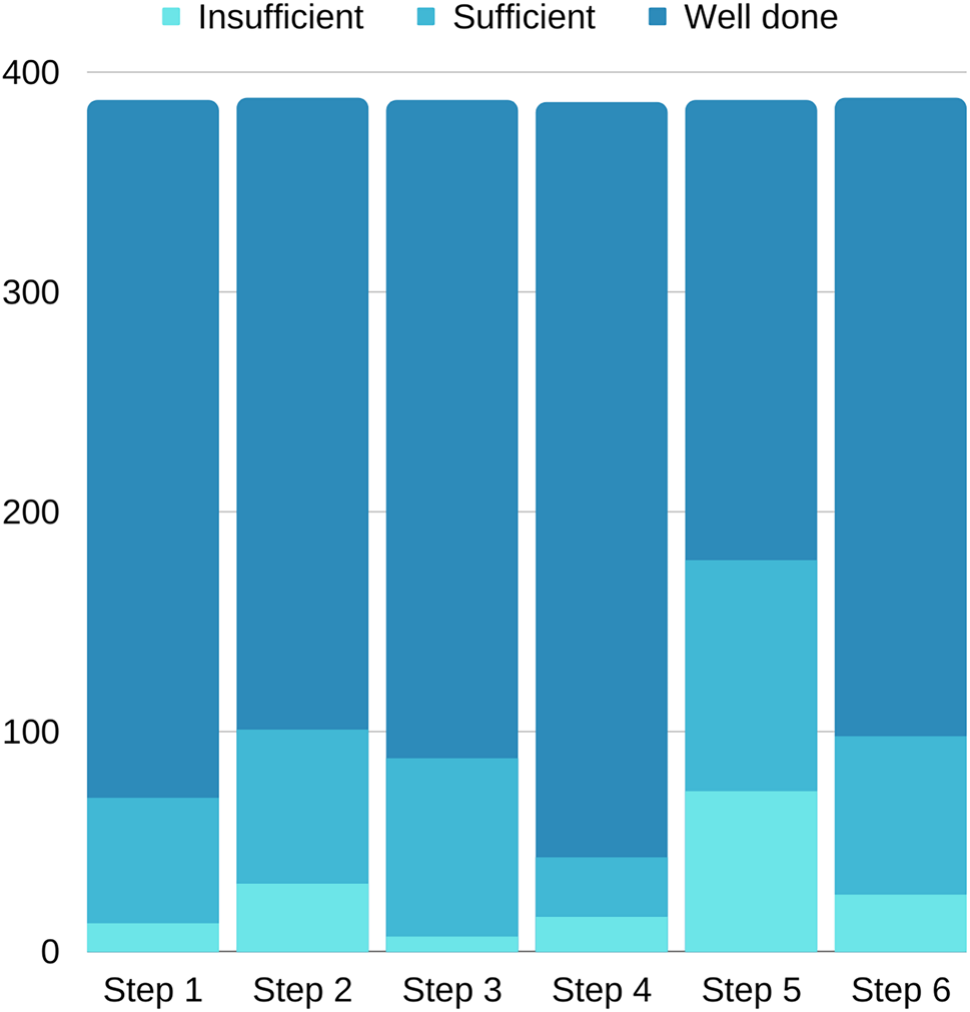
Scores on WHO six-steps. Step 1: define the problem. Step 2: specify the therapeutic objective. Step 3: specify the standard treatment. Step 4: choose a treatment suitable for the patient. Step 5: give information, instructions, and warnings. Step 6: monitor treatment

We analyzed the WHO six-step models made by all 388 students. Per step, the students could score insufficient, sufficient, or well done. Figure 2 shows that students score lower on step 5 in comparison to other steps.

### Repeated errors

There was a significant difference between error types in whether or not an error was repeated from the formative to the summative assessment (*P* < 0.001, Table 3). A total of 1249 errors were only made in the formative assessment and were not repeated in the summative assessment. Almost half of these errors (*n* = 591, 47%) could be assigned to the category missing information. Within this category, the subcategory of mentioning a child’s weight on the prescription (242 (41%)) was the most improved.

**Table 3:** Error types

	**Errors only in formative assessment (*n* and %)**	**Errors new in summative assessment (*n* and %)**	**Error repeated in both assessments (*n* and %)**
**Administrative** Opium Law	328 (26%) 190 (58%)	51 (10%) 9 (18%)	79 (27%) 15 (19%)
**Missing information**	591 (47%)	253 (49%)	145 (49%)
**Wrong drug dose**	75 (6%)	38 (7%)	21 (7%)
**Wrong drug interval**	62 (5%)	73 (14%)	14 (5%)
**Incorrect dosage form**	74 (6%)	14 (3%)	1 (0.3%)
**Incorrect prescribed amount**	114 (9%)	78 (15%)	33 (11%)
**Wrong drug**	5 (0.4%)	11 (2%)	1 (0.3%)
**Total**	1249 (100%)	518 (100%)	294 (100%)

Error types divided by (1) errors only made in the formative assessment, (2) errors newly made in the summative assessment, and (3) errors repeated in both assessments. % are all errors in the category divided by error type

Errors that were newly made in the summative assessment were errors, which the student did not make in the formative assessment and therefore did not receive feedback on. Most errors which were only made in the summative assessment (*n* = 82, 32%) were in the subcategory missing usage instructions, which fall in the overarching category missing information, see Table 4. An example of this category is forgetting to add that eardrops need to be used in the ear canal.

**Table 4:** Error type missing information

	**Errors only in formative assessment (*n* and %)**	**Errors new in summative assessment (*n* and %)**	**Error repeated in both assessments (*n* and %)**
Weight of child	242 (41%)	4 (2%)	8 (6%)
Concentration missing	17 (3%)	10 (4%)	1 (1%)
Dosage form missing	18 (3%)	0 (0%)	0 (0%)
Amount to supply missing	44 (7%)	8 (3%)	1 (1%)
Duration of treatment missing	61 (10%)	8 (3%)	0 (0%)
Maximum use missing	1 (0%)	31 (12%)	3 (2%)
Dose not measurable	25 (4%)	4 (2%)	1 (1%)
Wrong usage instructions	57 (10%)	37 (15%)	4 (3%)
Missing usage instructions	73 (12%)	82 (32%)	121 (83%)
“With controlled release” missing	8 (1%)	11 (4%)	0 (0%)
Dosage interval missing	1 (0%)	9 (4%)	0 (0%)
Confusing information	43 (7%)	49 (19%)	6 (4%)
Name of medication missing	1 (0%)	0 (0%)	0 (0%)
Total	591 (100%)	253 (100%)	145 (100%)

Error type missing information divided by (1) errors only made in the formative assessment, (2) errors newly made in the summative assessment, and (3) errors repeated in both assessments. % are all errors in the category divided by error typ﻿e

Finally, there were errors made by a student in both the formative and the summative assessment. Again, most of these were in the category missing information (*n* = 145, 49%). Of these errors, 83% (*n* = 121) were in the subcategory missing usage instructions.

### Feedback checked

We sent all 202 students who took their summative assessment between 17 May 2021 and 4 October 2021 a questionnaire on their preparation for the summative assessment. Of these, 71 (35.1%) students filled out the questionnaire. One student was excluded since the student did not take the formative assessment. These 70 students had an average age of 25.8 years, and 63% were female. This was comparable to the whole group of students (age *T*-test *P* = 0.248, gender chi-square test *P* = 0.666). To check whether this sample was representative of the whole group of students, scores of all students on the Dutch National Pharmacotherapy Assessment [19] were used to compare students who completed the questionnaire and the students who did not. The average score on the Dutch National Pharmacotherapy assessment did not differ between students who did and did not fill out the questionnaire (89.0% vs 89.4%, *P* = 0.24).

Of the 70 students who filled out the questionnaire, 63 (88.7%) students answered that they checked the feedback on the formative assessment in preparation for the summative assessment. Of these 63 students, 43 (68%) found the feedback useful in their preparation. Students mentioned that they felt well prepared and that they knew what was expected of them. The students who checked the feedback showed the same pattern of errors made in both assessments compared to the total group of included students, see Table 5.

**Table 5:** Errors made by students who checked the feedback on the formative assessment in preparation for the summative assessment

	**Errors only in formative assessment (*n* and %)**	**Errors new in summative assessment (*n* and %)**	**Error repeated in both assessments (*n* and %)**
**Administrative**	56 (29%)	18 (12%)	25 (29%)
**Missing information** Weight of child Missing instructions	87 (44%) 30 (34%) 16 (18%)	73 (48%) 4 (5%) 16 (22%)	37 (42%) 4 (11%) 24 (65%)
**Wrong drug dose**	17 (9%)	11 (7%)	10 (11%)
**Wrong drug interval**	7 (4%)	27 (18%)	5 (6%)
**Incorrect dosage form**	7 (4%)	1 (1%)	0 (0%)
**Incorrect prescribed amount**	22 (11%)	17 (11%)	9 (10%)
**Wrong drug**	0 (0%)	4 (3%)	1 (1%)
**Total**	196 (100%)	151 (100%)	87 (100%)

Errors made by students who checked the feedback on the formative assessment in preparation for the summative assessment. Error types divided by (1) errors only made in the formative assessment, (2) errors newly made in the summative assessment, and (3) errors repeated in both assessments. % are all errors in the category divided by error type

## Discussion

The aim of this study was to determine whether personalized feedback on a formative assessment helps medical students to increase their prescribing skills. In our study, we categorized errors from over 2300 prescriptions written by almost 400 medical students. This has shown for which category errors personalized narrative feedback can facilitate students’ learning.

Almost 46% of all errors that were resolved after receiving the personalized narrative feedback were based on basic patient safety issues. For example, this includes over 300 administrative errors and almost 250 errors now mentioning the weight of a child on a pediatric prescription, which is necessary for the pharmacist to be able to check the calculated dose.

On the contrary, errors that were repeated despite the feedback were largely based on developing the ability to empathize with a patient, to understand what information is needed on a prescription to have the patient execute the treatment correctly.

Our findings confirm the result of the study by Sabatino et al., where nurse practitioner students received formative feedback from pharmacists on assignments in which they had to identify errors in prescriptions and write a correct prescription [27]. Equal to our results, where the personalized feedback helped students to learn about the technical elements of prescribing, their students showed a greater increase in the performance of technical elements compared to the increase in the performance on clinical elements after a 14-week intervention with these weekly assignments.

This distinction between technical errors and errors made due to a lack of ability to empathize with the patient can also be seen in the results of the WHO six-step model. Steps 1, 2, and 3 focus on the indication, while steps 4 and 5 require the ability to put oneself in the place of the patient. While step 4, choose a suitable treatment for the patient, was often answered correctly, students had the most difficulty with step 5. In this step, students are asked what information they would communicate with their patient on instructions, efficacy, side effects, and warnings. This confirms the hypothesis that to train medical students in building this skill more frequent practice might be necessary, while this single formative assessment was able to help to increase technical elements in prescriptions.

In our study, the possible severity of the majority of the errors changed from a category B error (an error occurred but did not reach the patient) in the formative assessment to a category C error (an error occurred that reached the patient, but did not cause patient harm) in the summative assessment. This is not in line with the study by Lloyd et al. where pharmacist-led feedback on prescribing in a hospital setting showed no change in the distribution of error severity before and after feedback, but significantly reduced the frequency of all prescribing severity grades [12]. In our study, we have seen a decline in administrative errors after the formative assessment. These administrative errors are often categorized as a category B error, which makes the shift in error severity from category B to category C expected. In addition, this could be explained by the slightly increased difficulty of the cases asked in the summative assessment compared to the formative assessment.

Teaching medical students the skill of prescribing safely and effectively is a complex task. Our results show that personalized narrative feedback is a way to teach students how to write technically more correct prescriptions. However, even though Bertels et al. suggest that the personalized and individual way the feedback on this formative assessment is given is the preferred way [28], it does not seem to increase the gut feeling that students need to write prescriptions. Future studies should investigate if more frequent feedback on prescriptions during their education, compared to this one moment of feedback through a formative assessment, helps to increase this development.

There are some potential drawbacks associated with our study. For example, the summative assessment takes place one-and-a-half year after the formative assessment. This could mean that the results of our study are not only a direct result of the formative assessment, but due to other classes or practice time. However, all classes on the technical aspects of writing a prescription are given prior to the formative assessment. In addition, from the questionnaire, we know that students use the feedback given on the formative assessment in their preparation for the summative assessment, which makes a relation between the feedback given and the errors on the summative assessment plausible. Secondly, it could be discussed that students did not have the opportunity to make all errors in the formative assessment. While it is difficult to compare cases asked in both assessments, the teachers creating the assessments strive to equalize the difficulty of the cases between both assessments. Thirdly, we have not taken the quality of the feedback into consideration. It could well be that with an increased quality of the feedback students would have been able to increase their skills even more. Lastly, there was a relatively low response rate on the questionnaire, which could have biased the results. However, respondents’ scores on the knowledge assessment and the similar distribution of errors give the impression that the group might be representative of the whole group.

This is the first known study to examine the effect of a formative assessment on clinical pharmacotherapy education. A strength of our study was the number of prescriptions checked by a multidisciplinary team. Also, not only were the number of errors within both assessments checked, but it was also studied per student if errors were repeated or not. This made for highly detailed information on almost 400 students.

## Conclusion

Formative assessments not only assess students but can also ease students’ learning through feedback. Personalized narrative feedback can help students to increase the technical correctness of their prescriptions. However, errors repeated in the summative assessment are predominantly errors that show that this one formative assessment has not yet enhanced the clinical prescribing enough. Future research should concentrate on an intervention with more frequent personal feedback on the prescriptions of medical students.

## Supplementary Information

Below is the link to the electronic supplementary material.

Supplementary file1 (DOCX 148 KB)

## Data Availability

The datasets generated during and/or analyzed during the current study are available from the corresponding author upon reasonable request.
