# Supplementary material for: Does formative assessment help students to acquire prescribing skills?
Source: Eur J Clin Pharmacol. 2023 Feb 22;79(4):533–40. doi: 10.1007/s00228-023-03456-w (PMC9945830; doi:10.1007/s00228-023-03456-w)
Supplement: Supplementary file 1 — Supplementary file1 (DOCX 148 KB) [file 228_2023_3456_MOESM1_ESM.docx]

**Appendix 1 Example of an assessment in P-scribe; one prescription question and one WHO six-step question**


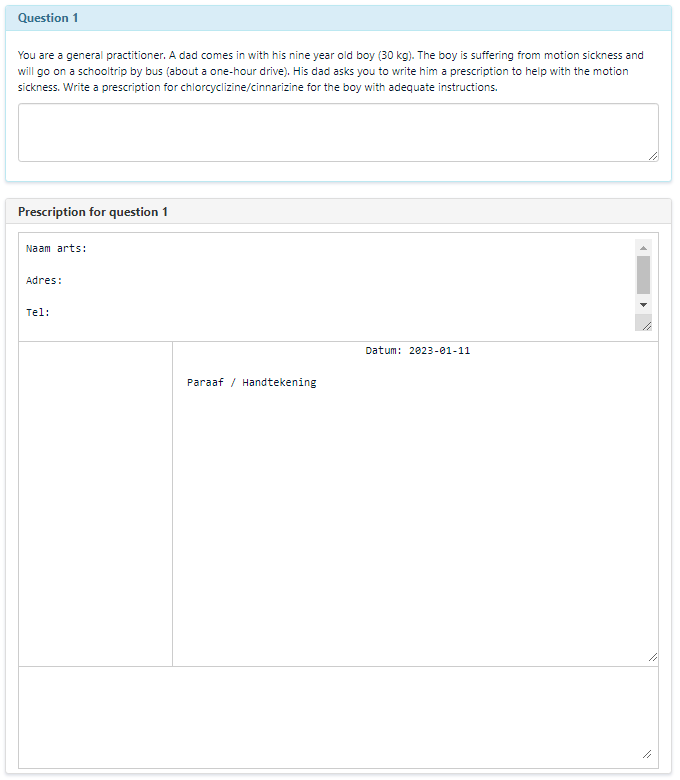


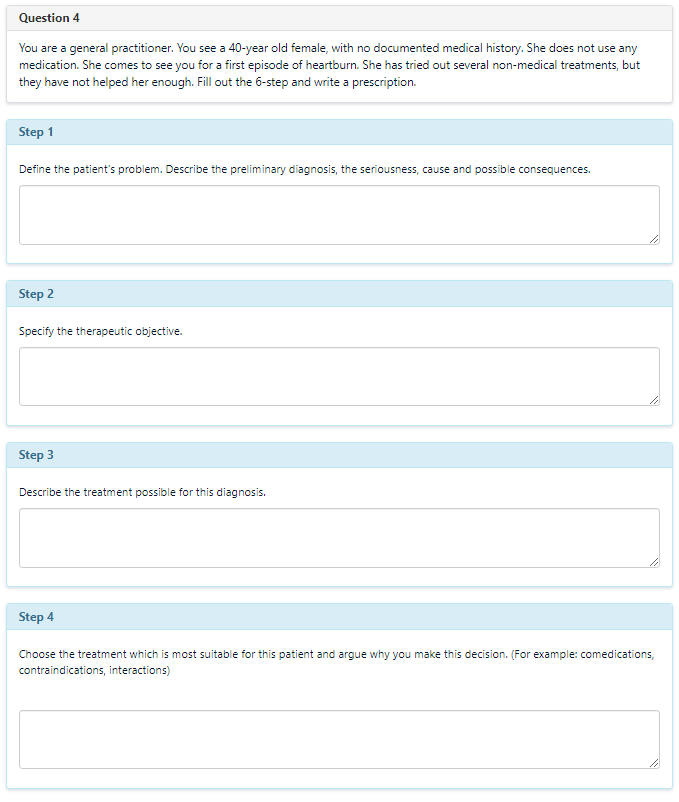


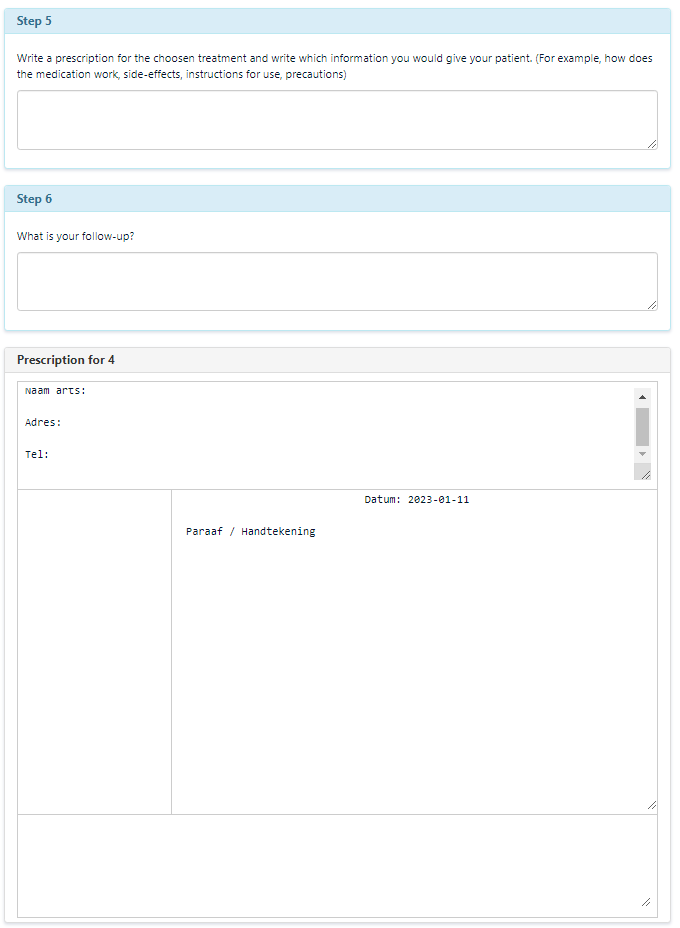


**Appendix 2; Visual of questionnaire after summative assessment**


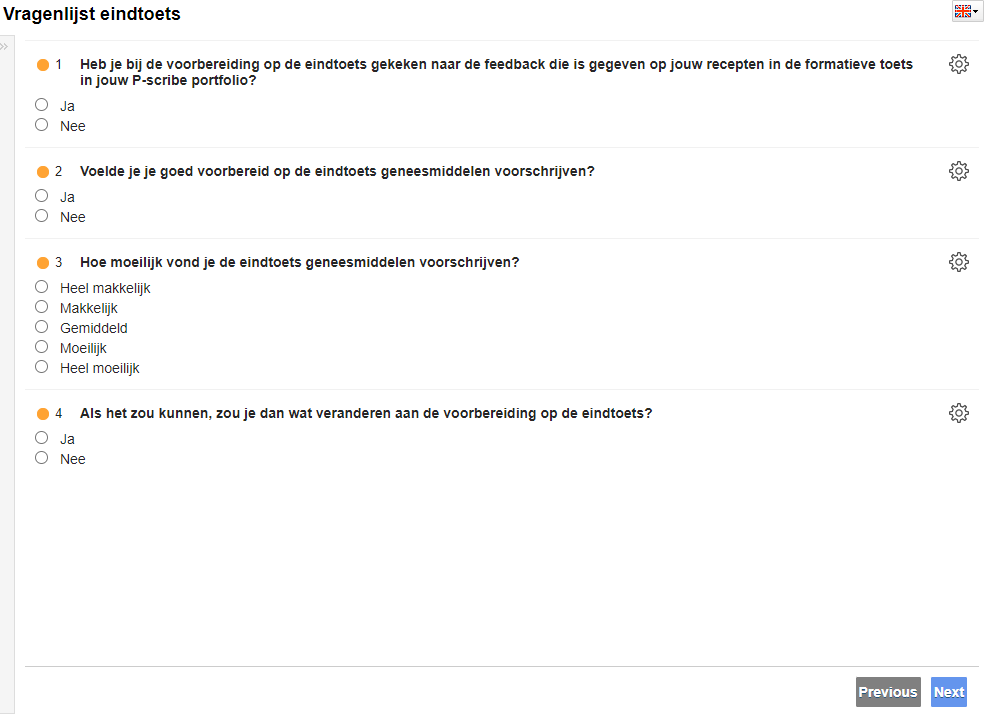


Translation:

Questionnaire summative assessment

1. In preparation for the summative assessment, did you check the feedback given on your formative assessment in your P-scribe portfolio? Yes/No
2. Did you feel well prepared for the summative assessment? Yes/No
3. How hard did you find the summative assessment? Very easy/easy/average/difficult/very difficult
4. If possible, do you want to change anything in the preparation on the summative assessment? Yes/No
